# The paradox role of caspase cascade in ionizing radiation therapy

**DOI:** 10.1186/s12929-016-0306-8

**Published:** 2016-12-07

**Authors:** Najmeh Rahmanian, Seyed Jalal Hosseinimehr, Ali Khalaj

**Affiliations:** 1 Department of Radiopharmacy, Faculty of Pharmacy, Tehran University of Medical Sciences, Tehran, Iran; 2 Department of Radiopharmacy, Faculty of Pharmacy, Mazandaran University of Medical Sciences, Sari, Iran; 3 Department of Medicinal Chemistry, Faculty of Pharmacy, Tehran University of Medical Sciences, Tehran, Iran

**Keywords:** Caspase, Radiotherapy, Cancer, Treatment, Induction

## Abstract

Radiotherapy alone or in combination with chemotherapy/surgery is widely used for treatment of cancers. It reduces tumor growth and prevents metastasis. While ionizing radiation activates caspase cascade resulted in apoptosis in cancer cells, it also stimulates tumor cell re-population that leads to reduce the effectiveness of the radiation therapy. This review describes the mechanisms for paradox role of caspase cascade in cancer therapy and discusses the logical and practical strategies for improvement the therapeutic index of radiotherapy through enhancement of radiosensitivity and decreasing the rate of tumor recurrence.

## Background

Radiotherapy is one of the helpful clinical procedure for treatment of different types of human malignancies especially lung, thyroid, breast, colon, prostate and brain cancers. Nearly 50% of all cancerous patients are receiving ionizing radiation (IR) at some stages of their treatments [1, 2]. Following irradiation, cancer cell death may occur mainly through necrosis, autophagy and apoptosis of which autophagy has more complex role and might promote cell survival [3–5]. IR prolongs patient’s survival through decreasing proliferative capacity and killing tumor cells [6–8]. However, re-population of tumor cells during or after radiotherapy is an important obstacle to achieve the desired response [9, 10]. Since death of high percent of tumor cell is a desirable response in radiotherapy regimen, identification of the limiting factors such as cellular proliferation and intrinsic radioresistance are very important in cancer treatment [6, 9, 11, 12]. Escaping from programmed cell death or apoptosis is one of the popular theories that explain cancer cell radioresistance [1, 13]. Caspase cascade as an apoptosis mediator in radiation therapy has been described to activate signal transduction pathways and expression of survival proteins [14]. In this review, the paradox role of caspase cascade in adjuvant therapy of the cancer by IR is discussed.

### Caspase cascade functions

Caspases (cysteine-aspartic proteases, cysteine aspartases or cysteine-dependent aspartate-directed proteases) are a family of protease enzymes that have critical roles in controlling homeostasis in apoptosis and inflammation processes [6]. On the basis of the mechanisms of actions, caspases have been categorized into initiators (apical: CASP2, CASP8, CASP9 and CASP10) and effectors (executioner: CASP3, CASP6, and CASP7). It is believed that caspases are present as inactive monomeric precursor enzymes that must be dimerized for full activation [11, 13, 15–17].

### Apoptosis

Apoptosis is a programmed cell death that is involving degradation of the cellular component such as nuclear DNA, the Golgi, endoplasmic reticulum (ER) and hydrolysis of mitochondrial networks by a group of cysteine proteases called caspase. A variety of stimuli including IR, chemotherapeutic drugs, death receptors-mediated processes like tumor necrosis factor α [TNFα], growth factor withdrawal, loss of cell adhesion (anoikis) and cytoskeletal damage might promote apoptosis pathway through activation of the caspase cascade [18].

#### Apoptosis inhibition

Caspase family have important roles in various diseases and it has been shown that caspase deficiency results in tumor development [19–21]. Suppression of caspase activation can be promoted by several proteins including: Bcl-2, Inhibitors of Apoptosis Proteins family (IAPs) and Cytokine Response Modifier A (CrmA) [22, 23]. Bcl-2 is an anti-apoptotic effector protein which prevents the distribution of pro-apoptotic proteins such as Bax in the mitocondria [24]. Human IAPs namely XIAP, c-IAPl, C-IAP2, NAIP, Livin and Survivin have been described [25]. CrmA prevents caspase dimerization for full activation. Generally, these groups of proteins inhibit cell death through the inhibition of caspase and suppression the activity of pro-apoptotic proteins like as Bax, Bad, Bim and Noxa and also by augmentation of the activity or expression of anti-apoptotic proteins like as Bcl-2, IAPs and CrmA [26].

#### Radiation-induced apoptosis

Following exposure to IR, reactive oxygen species (ROS) and free radicals are generated which induce DNA damages. Double-Strand Breaks (DSBs), is the most abundant and toxic DNA damage which results from the exposure to one Gy of IR [27]. Following of DNA damage, cell cycle arrest and DNA repair are activated. Also two important pathways namely ATM-CHK2 (Ataxia Telangiectasia Mutated-Checkpoint Kinase 2) and ATR-CHK1 axis (Ataxia Telangiectasia and Rad3-related Checkpoint Kinase 1) are activated and induced by DSBs and DNA single-strand breaks (SSBs), respectively [12]. These pathways have overlapping functions and act in parallel with each other [12, 28]. CHK2 and CHK1 phosphorylate different positions of the p53 which results in its dissociation from mdm2 (mouse double minute 2 homolog). P53 as a tumor suppressor has important role in initiation and prevention of the cancer. P53 has been named “guardian of the genome” because of its essential role in the selection of cell death or cell survival [29]. P53 activation regulates DNA repair processes and cell-cycle arrest that occurs at G1-S and G2-M transitions [3, 27]. Actually, the degree of DNA damage-repair is very prominent to determine whether cell survival or cell death [30]. Briefly, the p53 transcription factor responds to numerous cellular stresses and eliminates cells containing oncogenic lesions or damaged DNA, thus preventing tumor development [12]. Defective DNA repair results in promotion of aneuploidy and progression of cancer. The importance of p53 in sensitivity to radiation was discussed by several studies. For instance, resistance to radiation have been found in thymocytes lacking functional p53, whereas wild type p53 are extremely sensitive to radiation [31]. Moreover, radiosensitive apoptotic pathway is induced in colonic and leukemia malignant cells with overexpression of wild type p53 [32, 33]. In Summary, p53 expression has pivotal role in induction of p53-dependent apoptosis and cells with high p53 mRNA expression are more susceptible to radiation-induced apoptosis [34].

**Fig. 1:**
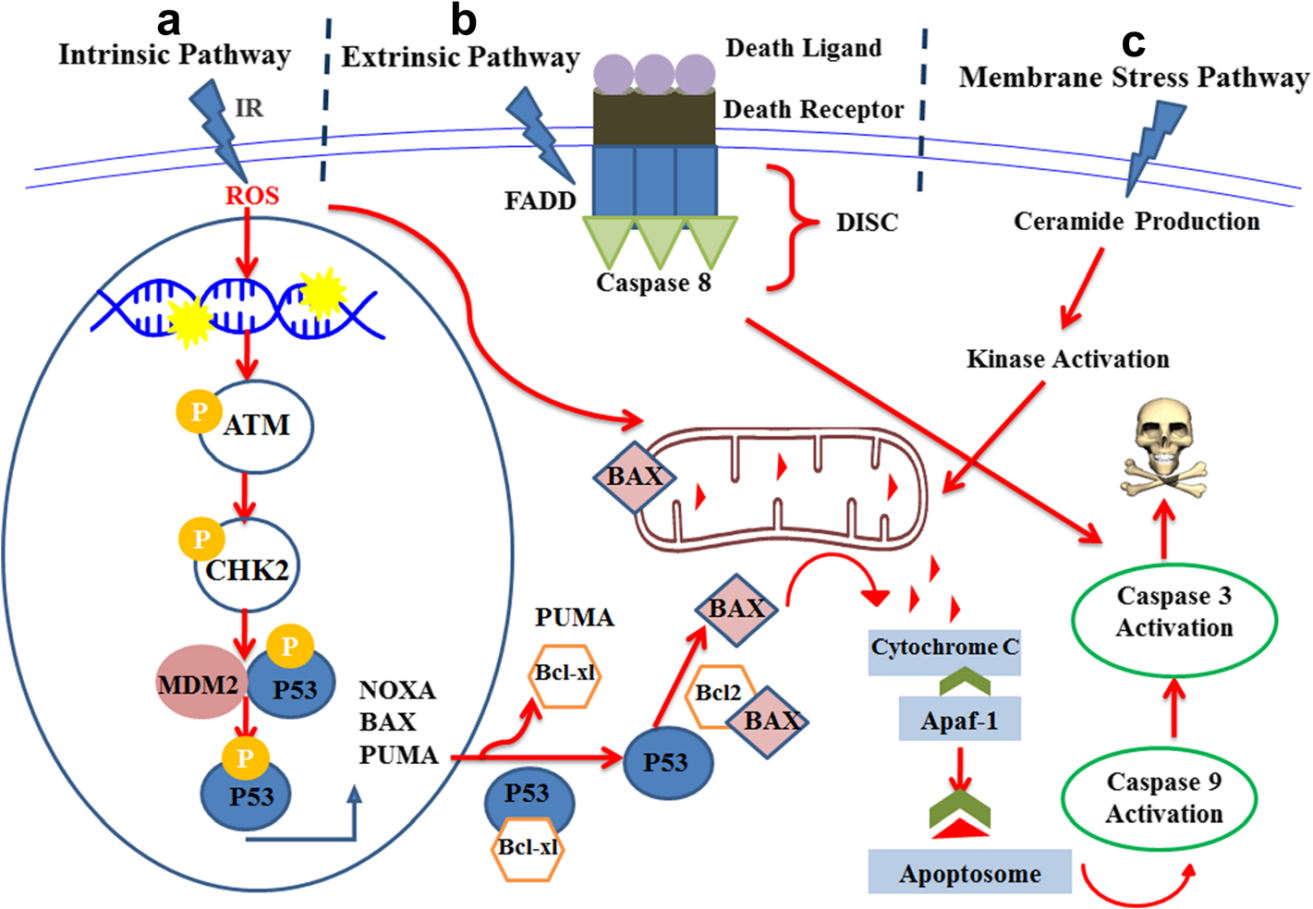
A summary of extrinsic, intrinsic and membrane stress apoptotic pathways. **a** DNA damages lead to activation of p53 that promotes the expression of pro-apoptotic proteins such as BAX and consequently permeabilization of the outer membrane of mitochondria and release of cytochrome C followed by formation of a large multieric complex namely Apoptosom through participation of cytochrome C, dATP and apoptotic protease-activating factor-1 (APAF-1). **b** Activation of p53 by ionizing radiation leads to downstream transactivation of death receptor and death ligand that trigger extrinsic apoptotic pathway followed by complex formation between death receptor and its cognate ligand. Complex formation results in receptor trimerization and consequently formation of death inducing signal complex (DISC) by participation of FADD (Fas-associated death domain) mediated by DD (death domain). **c** Apoptosis might be prompted by production of ceramide as a second messanger which is activated by double strand breaks (DSBs) and reactive oxygen species (ROS) and as a result activation of sphingomyelinase followed by hydrolysis of sphyngomyelin and release of ceramide. The outcome of apoptosis is caspase activation which results in cell death due to production of apoptosome, death inducing signaling complex (DISC) and ceramide in intrinsic, extrinsic and memberane stress apoptotic pathways, respectively

IR frequently induces intrinsic (mitochondrial mediated), extrinsic (death receptor mediated) and membrane (ceramide production) apoptotic pathways. Intrinsic and extrinsic apoptotic pathways are activated by p53 and membrane stress pathway is promoted by contribution of ceramide as a second messenger (Fig. 1) [35].

#### Extrinsic apoptotic pathway

In the extrinsic apoptotic pathway, a complex is formed between the death receptors such as CD95 (APO-1/Fas), TNF receptor 1 (TNFRI), TNF-related apoptosis-inducing ligand-receptor 1 (TRAIL-R1) and extracellular ligands including CD95 ligand (CD95L), lymphotoxin-α and TNF-related apoptosis-inducing ligand (TRAIL-1). It should be noted that activation of p53 by radiation causes downstream transactivation of both death receptors and ligands [35, 36]. Complex formation between the death receptors and their cognate death ligands results in receptor trimerization and clustering of the death domain receptors. Also, recruitment of an adaptor protein like as fas-associated death domain (FADD) mediated by Death Domain (DD) lead to the formation of the death inducing signaling complex (DISC) by interaction between pro-caspase 8 and FADD. The resulting complex activates caspase-8 as a caspase initiator which in turn triggers executioner caspases 3 and 7 and as a consequence results in the cell death [17, 35].

#### Intrinsic apoptotic pathway

Apoptotic mitochondria pathway is regulated by Bcl-2 family. Following p53 activation in response to a stress such as IR, PUMA (p53 Upregulated Modulator of Apoptosis) expression is results in the release of BAX a member of the Bcl-2 family. The Bcl-2 family may act either as anti-apoptotic (e.g. Bcl-2, Bcl-xl) or pro-apoptotic (e.g. Bax, BAD). Bax expression leads to Mitochondrial Outer Membrane Permeabilization (MOMP) that results in release of the cytochrom C from mitochondria intermembrane space to cytoplasm [35]. Moreover, IR may increases the formation of O_2_^•^ in mitochondria directly which in turn triggers the release of cytochrome C [37]. Results of the recent studies suggest that cytochrom C, dATP and an adapter protein namely apoptotic protease-activating factor-1 (APAF-1) are involving in the formation of a large multimeric complex called apoptosome. Activation of the procaspase 9 through with apoptosome leads to the cell death.

#### Membrane stress pathway

In contrast to intrinsic and extrinsic pathways, membrane stress pathway is independent of p53 [38]. Release of Ceramid as a second messenger is necessary to initiate this pathway. Ceramid can be formed through two different ways. Oxidative damages of ROS on lipid membrane results in activation of sphingomyelinase which in turn hydrolyze sphingomyelin of the membrane to ceramide [35, 39]. Also the high dose of IR induces DSBs which activates the synthesis of ceramide [40]. Ceramide promotes MAP/ERK (Mitogen Activated Protein/Extracellular Signal-Regulated Kinase) (MEKK) pathway which in turn activates Mitogen-Activated Protein Kinase 8 (MAPK8) and of the effector caspases-1, -3 and -6 that promote the cell death pathway [35].

### Suppression of radiation-induced apoptosis

IR induces multiple pro-survival signaling pathways through activation of ERK, c-Jun N-terminal Kinase (JNK), p38 MAPK and overexpression of COX-2 that can suppress apoptosis and reduce the cytocoxicity of radiation in radioresistance cancer cells. The mechanisms of these survival signaling pathways induce cell viability and inflammation are as follows:

#### Radiation- induced ERK1/2 pathway

IR activates human epidermal growth factor receptor (HER) signaling pathway which consists from four plasma–membrane bound receptor tyrosine kinase HER1, HER2, HER3 and HER4 [41]. IR mimics the behavior of EGF and induces HER dimerization which is followed by autophosphorylation of several tyrosine residues in the c-terminal of the regulatory tail of the receptor. HER activation stimulates Ras/Raf/MEK/ERK pathways through the exchange of GDP bound to Ras for GTP [35]. MEK activates ERK1/2 through phosphorylation of threonine and tyrosine which in turn inactivate apoptosis through inhibition of some pro-apoptotic proteins such as Bim, Bad, caspase 9. It also enhances expression of anti-apoptotic proteins such as Mcl-1, Bcl-xl. In summary radiation-induced activation of ERK1/2 signaling results in apoptosis suppression in irradiated cell by enhancing the activity/expression of anti-apoptotic proteins such as Bcl-xl and prevention of the activity of pro-apoptotic proteins [42–45].

#### Radiation-induced Akt signaling pathway

Upon HER hetrodimerization and autophosphorylation, PI3K/AKT1 and Ras/Raf/MAPK pathways are activated. AKT is a vital pro-survival factor that suppresses the apoptotic signaling pathway and leads to activation of pro-survival pathways and cell development [46]. Following HER dimerization and autophosphorylation of tyrosines in the regulatory intracellular domains of the receptor, six docking sites are formed [47]. Binding of the p85 regulatory subunit of the class 1 phosphatidylinositol 3-kinases (PI3K) to docking sites recruits the p10 subunit of PI3K as a catalytic subunit to form fully activated phosphatidylinositol 3-kinase (PI3K) enzyme. PI3K converts phosphatidylinositol-4,5-biphosphate (PIP2) to phosphatidylinositol (3,4,5)-triphosphate (PIP3) which activates phosphoinositide-dependent kinase (PDK) [48]. AKT is activated at first through phosphorylation of Thr 308 present in its kinase domain by activated PDK1 and then by phosporylation of Ser 473 present in its C-terminal domain by PDK2 [42, 48]. Activated AKT negatively regulates apoptotic pathways at least through seven mechanisms. I) phosphorylation and inhibition of the pro-apoptotic proteins of Bcl-2 family (e.g. Bad, Bax, Bim) [46] and inhibition of the expression of the pro-apoptotic proteins (Bim, Noxa) [49], II) phosphorylation of NF-_К_B as a pro-survival transcription factor that leads to expression of the anti-apoptotic proteins including Bcl-2 and Bcl-xl. [50], III) activation of XIAP (X-linked inhibitor of apoptosis protein) as a pro-survival protein that inhibits caspase 3, 7, 9 and results in suppress apoptosis [51], IV) activation of m-TOR kinase signaling pathway and other pro-survival signaling pathway that results in activation of mcl-1 as an anti-apoptotic protein [52], V) direct inhibition of the pro-apoptotic transcription factor Foxo3a as a positive regulator which induces expression of pro-apoptotic proteins [53], VI) phosphorylation of glycogen synthase kinase which results in inhibition of apoptotic induction by this enzyme under hypoxia during radiation therapy [54], VII) direct activation of non-homologous end joining (NHEJ) as a pathway which repairs DSBs damages and results in enhanced cell survival [55]. In summary, all the above mentioned mechanisms can be activated by AKT signaling pathway, which results in cancer cell development in response to radiation [42].

#### Radiation-induced JAK-STAT signaling pathway

IR activates JAK-STAT signaling pathway by increasing in the phosphorylation of Janus-Associated kinase 2 (JAK-2) and consequently Signal Transducer and Activator of Transcription (STAT). Translocation of STAT to the cell nucleus increases the levels of Bcl2/Bcl-xl proteins that are leading to decrease caspase-3 activity, tumor survival and radioresistant lung cancer cells. Niclosamide as an effective STAT3 inhibitor has shown to block effectively STAT3/Bcl2/Bcl-xl and to reduce the radioresistance in animal lung cancer xenografts [56, 57].

#### Radiation-induced Cox-2 overexprresion

Cyclooxigenases or prostaglandin synthases (COXs or PGH synthase) also known as COX-1, COX-2 and COX-3 are required enzymes for the formation of prostanoids including prostaglandins and thromboxane. Of three isozymes, COX-2 has aggressive behaviors in tumors and is overexpressed in a variety of malignant tumors such as melanoma and breast cancer [58, 59]. Moreover, IR increases the production of COX-2 proteins. Several pro-survival pathways are mediated by COX-2 and result in tumor progression. Prostaglandin E2 (PGE2) is one of the main products of these pathways that has important role in cell proliferation, cell migration, tumor invasion and cell death [56]. PGE2 increases intracellular cAMP levels which activates protein kinase A (PKA) and phosphatidylinositol 3-kinase (PI3K) enzymes. PGE2 increases the level of Bcl-2 in adenocarcinoma and activates MAPK pathway through transactivates EGFR by increase in amphiregulin levels as a well-known EGFR ligand [56, 60, 61]. Yamakie et al. reported that PGE2 activated sarcoma kinase in non-small cell lung cancer through phosphorylation and activation of STAT3 [62]. Briefly overexpression of COX-2 in cancer cells mediates several pro-survival pathways including; MAPK/ERK, JAK/STAT, PI3K/AKT that enhance expression of pro-survival proteins such as NF-_К_B, Bcl-2 and Bcl-xl, IAP. It inhibits expression of pro-apoptotic proteins including Bad, Bax, and Bim that lead to inactivate caspase cascades and subsequently suppress apoptotic pathway. Therefore it seems that COX-2 inhibitors such as celecoxib might be useful to enhance the therapeutic index of chemoradiation through sensitization of tumor cells and as well as protection of normal cells to IR [56, 60, 61]. Radiosensitivity effect of COX-2 inhibitors will discuss in next section.

### Activation of radiation–induced apoptosis: caspase inducers

It is well known that the defects in caspase cascade activation lead to tumor progression and metastasis while its activation by compounds sensitizing tumor cells to IR result in induction of apoptosis, reduction in tumor size and control of cancer development. The amplification in expression of CASP3 protein after exposure to radiation induces the cell death through apoptotic pathway [63, 64]. It has been demonstrated that apoptotic receptor agonists such as monoclonal antibody ligands that bind specifically to TRAIL receptors (TNF-related apoptosis-inducing ligand) in combination with IR cause a synergistic apoptotic effect through the activation of caspase cascade pathway [65–68].

#### Radiosensitizers

Compounds known as radiosensitizers enhance therapeutic index of radiotherapy through inducing apoptosis. One of the most important reasons for the resistance to chemotherapy and /or radiotherapy is the presence of hypoxic cells in solid tumors because of insufficient blood supply to these tumors. Oxygen by formation of free-radical DNA damage increases the effectiveness of radiation and it is known as a potential radiosensitizer. Nitoaromatic and nitroheterocyclic compounds act as radiosensitizers by formation of nitro-anion radicals like oxygen. The synthesis and in vitro cytotoxicity of several dinitrophenyl derivatives of 5-fluorouracil [69, 70], 3-[(2,4-dinitrophenylamino)alkyl] derivatives of 5-fluorouracil [71], metronidazole tethered 5-fluorouracil [72] as radiosensitizers have been described. These compounds had little or no aerobic cytotoxicity while showed high cytotoxicity and radiosensitizing effect under hypoxic conditions.

The radiosensitizing effects of luteolin have been investigated in H1299 and NCI-H460 non-small cell lung cancer. Results have shown that inhibition of the cyclin-dependent kinase 2 by luteolin led to the cell-cycle arrest and as a consequence activation of the apoptosis. Also luteolin could inhibit the function of Bcl-2 in B-cell lymphoma 2 as a pro-apoptotic protein inhibitor and promote activation of caspase-3, -8 and -9. It has been reported that combination of radiation and luteolin increased NSLC cell deaths and decreased tumor growth in nude mice bearing NCI-H460 cell xenograft [73]. Luteolin remarkably enhanced the radiosensitivity of gastric human tumors by a significant downregulation of PGE2 production and augmentation of the caspase activity simultaneously [74]. Plumbagin a naphthoquinone derivative acts as a radiosensitizer in numerous cancer cell lines like melanoma and cervix cancer. The combination of plumbagine and 2 Gy of radiation has shown synergism effects and was more effective in induction of cell deaths as compared to 10 Gy of radiation alone. Plumbagine activates caspase 3 and suppresses Bcl-2 and Bcl-x as anti-apoptotic molecules without any changes in expression of Bax as pro-apoptotic protein. In summary, the combination use of sensitizing agent and IR has more effective in cell growth inhibition as compared to the use of only higher dose of IR [75].

#### Neutralization of IAPs (inhibitors of apoptosis proteins)

**Fig. 2:**
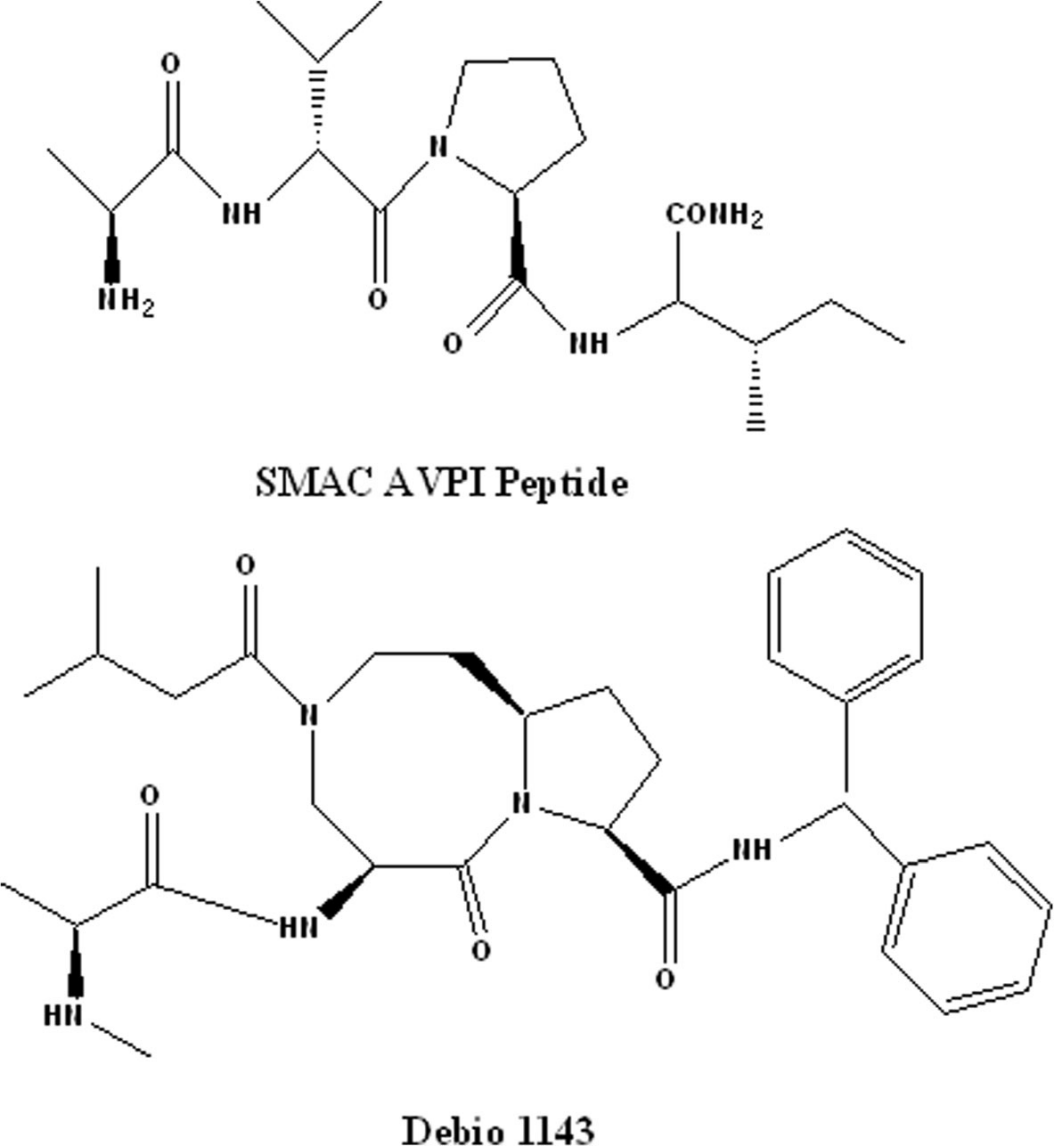
Chemical structures of Smac AVPI peptide (Second mitochondria-derived activator of caspase Ala-Val-Pro-Ileu) and Debio 1143

Recently several approaches for the cancer treatment are proposed in induction of apoptosis and increases the radiosensitizing effects through neutralization of IAP. One of the most important strategies for stimulation of caspase activation and sensitization of cancer cells to apoptosis is design and development of a new class of compounds mimicking endogenous IAP antagonists. By stimulation of apoptosis second mitochondria-derived activator of caspase (Smac) also known as DIABLO (direct IAP binding protein with low pI) is released from mitochondria into cytosol that antagonizes IAPs as XIAP, cIAP1 and cIAP2 and also Smac stimulates the activation of caspase 3 and subsequently promotes apoptosis pathway. Ala-Val-Pro-Ile (AVPI) tetrapeptide in Smac /DIABLO competes directly with a similar tetra peptide, Ala-Thr-Pro-Phe (ATPF) motif present in caspase 9, and by binding to a domain in XIAP prevents its binding to caspases 3, 7 and 9 (Fig. 2) [76]. It has been proposed that the combination use of a Smac mimetic and radiation may sensitize cells to killing effects of radiation through neutralization of the activity of IAPs that result in overcomes apoptosis, causes the release of cytochrome c and triggers caspase-3 activation in the following of apoptosome complex formation [77–79].

**Fig. 3:**
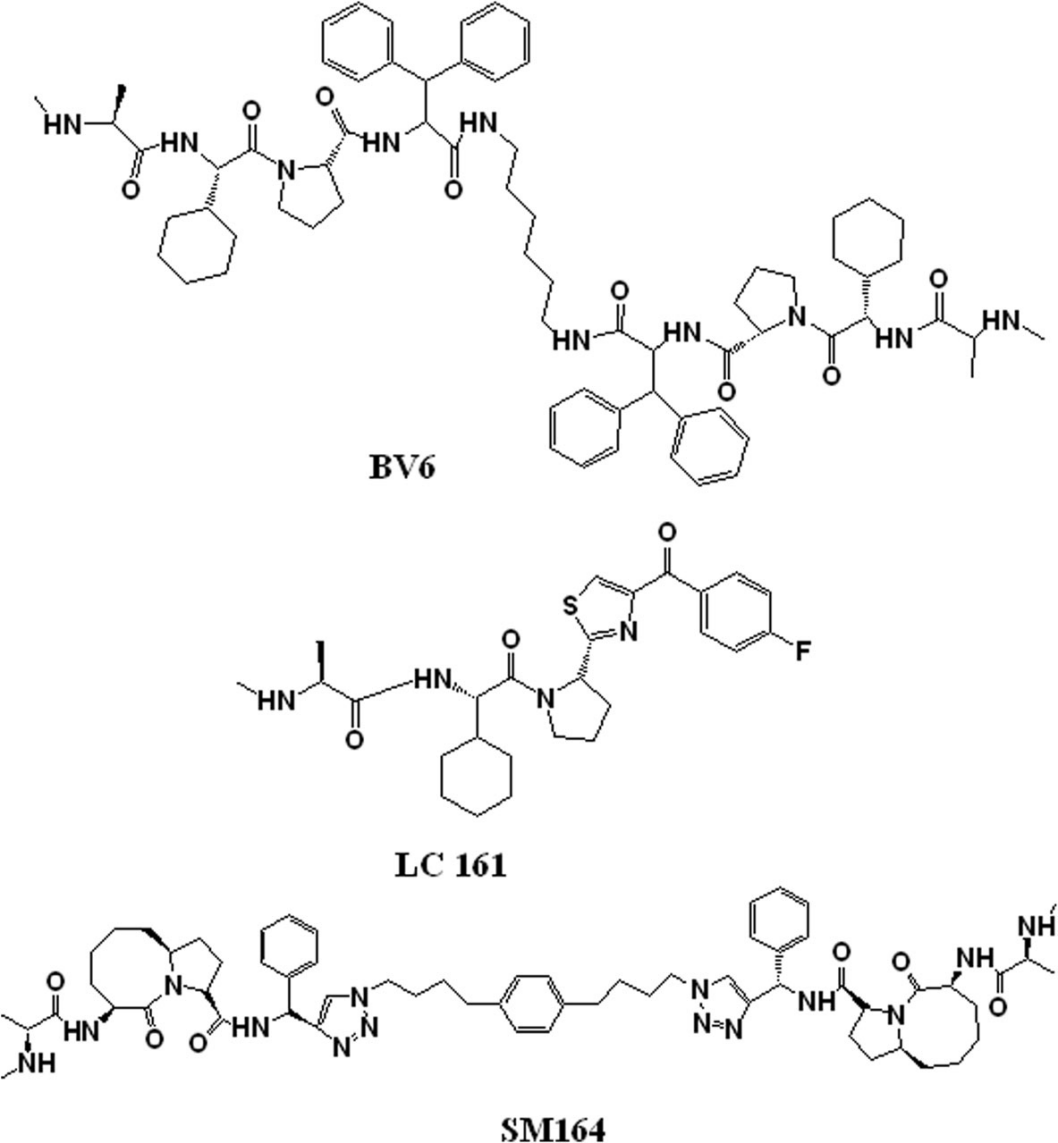
Chemical structures of Smac mimetics BV6 (1), LC 161 (2) and SM164 (3)

Recently several Smac mimetics have been introduced to neutralize IAP proteins and eliminate resistance of lung, breast, prostate and colorectal carcinoma to IR [79]. For example, small molecule Smac mimetic Debio 1143 or SM-406 were shown to be a potent sensitizers of NSCLC cells to effects of IR-induced apoptosis through cleavages of caspasse-3, -8 and a decrease in IAPs level. Debio 1143 remarkably enhanced the radiosensitivity by autocrine TNF-α production in cancer cell lines (Fig. 2). It has been shown that the release of TNF-α activates bindings of caspases 3 and 8 to TNFRs and provokes interaction of the Fas-associating protein with a death domain (FADD)/ receptor interacting protein 1 (RIP1)/RIP3 which results in the death of cells by promotion of the apoptosis pathway. Furthermore it has been shown that the radiosensitization effects could be potentiated by increase in the drug concentration, incubation time and TNF-α stimulation [80]. A number of XIAP antagonists with nanomolar affinity to the baculovirus IAP repeat 3 (BIR3) domain of XIAP have been described which promote apoptosis in resistance cancer cell lines and inhibit the growth of tumors in a MDA-MB-231 breast cancer mouse xenograft model [81]. Also it has been reported that the use of XIP antagonists increase the efficacy of radiotherapy by a significant decrease in the viability of gliobelastoma cells and increase γ-irradiation induced apoptosis by enhancement in cleavage of caspases 3 and 8 into active fragments [82]. BV6 is another small Smac mimetic-molecule that is radiosensitizer. BV6 is able to sensitize several glioblastoma cell lines to effects of radiation by stimulation of NF-kB activation, γ-irradiation–triggered caspase activation, cytochrom C release and increase in mitochondrial outer membrane permeabilization (MOMP) (Fig. 3) [83, 84]. It has been shown that the Smac mimetic LCL161 could increase the efficacy of radiotherapy in esophageal squamous cell carcinoma through the activation of caspase 8 and down regulation of IAP expression (Fig. 3) [85]. The efficacy of Smac mimetic SM164 in sensitization of breast carcinoma and head and neck squamous cells to effects of IR has been demonstrated (Fig. 3) [86–88]. Mechanistic studies have revealed that exposure of carcinoma cells to SM164 and radiation results in induction of apoptosis by activation of caspase cascade pathway and inhibition of IAP [86].

Survivin is a unique member of IAP family that acts as an inhibitor of caspase cascade. High expression of survivin results in suppression of apoptosis which is strongly correlated with radioresistance in most human tumor cells. It has been suggested that inhibition of survivin results in radiosensitization in treated cancerous cells [89–91]. For instance, treatment of human epidermoid carcinoma cell line A431 with adenoviral-mediated wild-type p53, as a survivin antisense, resulted in radiosensitivity [92]. Attenuation of survivin expression is also reported by treatment of human melanoma cells with a hammerhead ribozyme. Ribozyme expressing cells which have high sensitivity to effects of gamma radiation catalytic activity [93]. When siRNA was applied for downregulation of survivin expression in sarcoma and pancreatic cancer cell lines, there was an increase in caspase activity followed by a significant decrease in radioresistance. As a result this combination therapy perhaps be useful for the treatment of sarcoma and panceratic resistance carcinoma [94, 95].

Oxaliplatin is an anti neoplastic drug that is used for treatment of colorectal cancer. The combination of oxaliplatin and radiation is another logical strategy to control proliferation of the head and neck squamous cell carcinoma (HNSCC) by diminishing survivin expression and as a result induction of apoptosis through enhancement in caspase-3 activity. Oxaliplatin treatment markedly decreased expression of survivin in cancer cells. The cytotoxicity of oxaliplatin and radiation combination was greater than IR or oxaliplatin alone [96].

#### Radiosensitizing effect of COX-2 inhibitor agents

Several studies demonstrated that COX-2 has a key role in cell proliferation in cancer cells because its expression altering function/regulation of pro-apoptotic proteins. It is cleared that COX-2 selective inhibitor is correlated with better the tumor growth control and response to radiation [97]. A well-known mechanism for radiosensitivity activity of COX-2 inhibitor e.g celecoxib is induction of apoptosis through reduction of activity/expression pro-survival proteins such as survivin, Bcl-2, Bcl-xl, upregulation the level of P53, amplifying G2/M phase arrest, activation of intrinsic and extrinsic pathways of apoptosis and increase the level of expression/activation of caspase 8, caspase 9 [56, 60]. Grimes et al. showed nimesulide, a COX-2 selective inhibitor, has radiosensitivity activity in A549 non-small cell lung cancer cells and in nude mice bearing A549 tumor xenografts. Nimesulide inhibited NF-κB activity, with anti-apoptotic effect, that led to down-regulate the superoxide dismutase (MnSOD) with anti-oxidant activity, and surviving. Combination treatment with nimesulide and 30 Gy fractionated radiation resulted to diminish in tumor size compared with radiation only [98]. In esophageal squamous cell lines TE2 and T.Tn, the combination of radiation and celecoxib enhanced the radiotherapy efficacy through arresting the G2/M phase, inhibiting the expression of prostaglandin that has radio-protective effect. Celecoxib inhibited the DNA repair process and enhanced the expression/activation of caspase 8 and caspase 3 [99]. Radiosensitivity effect of diclofenac in COX-2 overexpressed human prostate adenocarcinoma (LNCaP-COX-2) and the control cell (LNCaP-Neo) was evaluated. LNCaP-COX-2 cells were more resistance to IR than LNCaP-Neo cells. Their results confirmed the radiosensitivity effect of diclofenac due to reduction the survival fractions at 2 Gy in presence of diclofenac (35.5%) in comparison with radiation only (78.6%) in LNCaP-COX-2 cells. Also, insignificant differences of radiosensitivity were found in LNCaP-Neo cells treated with diclofenac. Diclofenac has synergistic effect with IR through induction TRAIL-induced cell death that enhances the activation of caspase 8, caspase 9 and caspase 3. Delay in tumor growth was observed in combination of topical diclofenac gel and radiation than either radiation or diclofenac only [100]. Decrease the level of COX-2 expression by NS-398 as a selective COX-2 inhibitor led to amplyfing G2/M phase arrest in irradiated melanoma cell lines WM35 and LU1205 and subsequently decrease in survival of melanoma cells [101]. The radiosensitivity effect of NS-398 was shown in human prostate carcinoma cells PC3 with expressing COX-2, not radiosensitivity effect was seen in COX-2 knockdown PC3 cells [102]. It was shown that curcumin induced radiosensitivity and apoptosis by inhibition of survivin and COX-2 and result to activation of the caspase 3 and down-regulation of prostaglandin E2 simultaneously [103–105]. The potential role of COX-2 inhibitor to enhance the efficacy of radiotherapy was reviewed recently [56].

**Fig. 4:**
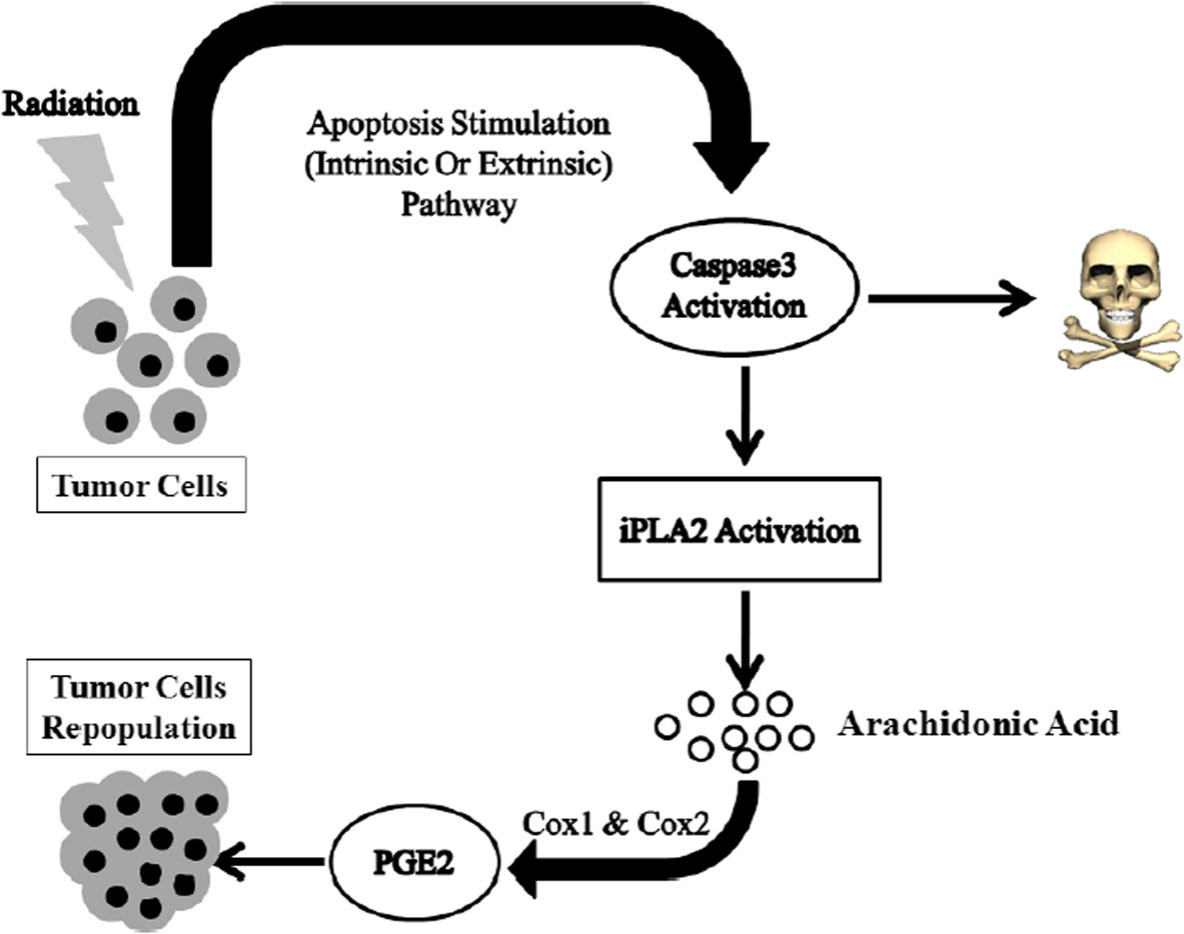
A schematic representation of caspase activation resulting in cell death and tumor cell repopulation. Caspase 3 upregulation followed by apoptosis stimuli lead to iPLA2 (Ca(2+)-independent cytosolic phospholipase A2) activation and cell death. Hydrolysis of fatty acids by iPLA2 results in arachidonic acid release and production of PGE2 (Prostaglandin E2) a well-known product of cyclooxygenase enzymes which induce tumor cell repopulation

Results of several studies have shown that induction of the caspase cascade pathway by radiotherapy other than enhancement of sensitization of tumor cells to effects of radiation might results in tumor cell re-population that will be discussed in the next section (Fig. 4) [14, 106, 107].

### Radiation-induced tumor cell repopulation: caspase 3-dependent activation of PLA2

Phospholipazes A2 are membrane enzymes that hydrolyze acyl esters of phospholipids at the sn-2 bond to produce arachidonic acid and lysophospholipids. These enzymes are commonly classified into secreted, cytosolic, lipoprotein-associated and Ca ^2+^ independent PLA2s [108, 109]. Of these enzymes the Ca ^2+^ independent PLA2s have numerous biological activities such as cell differentiation, cell proliferation, and cell death and it has been reported that changes in iPLA2 expression results in many disorders [108–110]. Results of investigation on human PLA2 structure have shown that this peptide has 806 amino acid, contains an ankyrin-repeat domain, a putative caspase-3 cleavage motifs and a catalytic site [109]. Cleavage of iPLA2 at Asp83 of TNFα/cycloheximide-treated U937 cells results in excessive release of fatty acids which implies caspase-3-mediated activation of iPLA2 [109, 111]. This finding is important because induction of apoptosis by activation of caspase cascade might result in migration and proliferation of malignant cells. As an example it has been shown that caspase 3 facilitated growth of malignant cells by overexpression of iPLA2 [112]. Furthermore repopulation of surviving cancer cells, as a common obstacle, during the intervals between two doses of radiotherapy is mediated by caspase 3 [14]. It has been reported that upregulation of caspase 3 which is induced by radiation other than acting as an effector to mediate apoptosis, increases significantly recurrence of cancers and deaths. It has also been shown that the cells with high expression of executioner caspases cause wound healing and tissue regeneration through a pathway called “Phoenix Rising” [113, 114]. Actually activation of iPLA2, that could be caspase-3 mediated, generates PGE2 and promotes tumor repopulation (Fig. 4) [14, 113]. Molecular studies have shown that recruitment macrophages that govern elimination of apoptotic cells after radiotherapy induce tumor repopulation by production of a clearance-related cytokine milieu including PGE2 [106, 107, 115]. Results of a recent study showed that after radiotherapy, the increase in caspase 3, caspase 7 and protein kinase Cd (PKCd) levels agitate the proliferation of surviving pancreatic tumor cells [106]. A remarkable reduction in recurrence of living cell as compared to wild-type pancreatic cells has been observed in combination therapy involving the use of radiation in dominant-negative mutants of caspase 3 (DN_C3), caspase 7 (DN_C7), or PKCd (DN_PKCδ) [106].

#### The effect of autophagy stimulators on tumor growth

Autophagy induction is one strategy for reducing tumor cell repopulation and enhancement of cell death in cells non-expressing apoptotic regulatory proteins or resistance to multiple apoptotic stimuli [116, 117]. Autophagy is another form of cell death that could negatively (e.g. mammalian target of rapamaycin (mTOR), Bcl-2) or positively (e.g. Beclin, ULK complex (Unc-51-like kinases) regulate [118]. Irradiation of Bax^-^/Bak^-^ mouse embryonic fibroblasts (MEF) cells were accompanied with enhances the level of pro-autophagy proteins such as Becline-1 and ATG5-ATG12 complex. A markedly radiosensitizing effect was observed in Bax^-^/Bak^-^ MEF cells that was mediated through autophagy induction. No radiosensitization response was found in MEF cell treated with 3-methyladenine (3-MA) as an inhibitor of autophagy. The radiosensitization effect was increased in cells treated with Rad001 as a mammalian target of rapamaycin (mTOR) inhibitor [119]. A significant enhancement in radiation efficacy was found in prostate cancer cells PC-3 and DU-145 treated with combination rapamaycin (mTORI) and Z-VAD (a caspase inhibitor) with IR [120]. A combination of Z-DEVD (a caspase-3 inhibitor) and radiation has been reported to enhance radiosenstization in a mouse with lung cancer by induction of autophagy pathway [115]. Delay in tumor growth and cellular proliferation (Ki67 staining), reduce angiogenesis (vWF staining) and apoptosis (TUNEL staining) confirmed this radiosensitizing effect that was mediated through induction autophagy. In summary, recruit of caspase inhibitor with radiation to stimulate autophagy pathway may be a useful strategy to increase survival times of patients and a remarkable delay in tumor recurrence.

#### The effect of dual COX-2 and EGFR inhibitor on tumor growth

As previously discussed, there is a cross talk between dual COX-2 and EGFR pathways. Briefly, PGE2 could transactivate EGFR with several complex processes such as enhancement the expression of amphiregulin, AKT, ERK and sarcoma dependent pathways [60–62]. On the other hand, EGFR could enhance COX-2 expression in normal and tumor cells such as head and neck squamous cell carcinoma (HNSCC) cell line mainly through Ras/Raf/MAPK [121, 122]. Several studies suggested that combination treatment with EGFR and COX-2 inhibitor led to more antiangiogenic and antitumor activity with supra-additive reduction in the levels of multiple pro-survival proteins such as p-ERK1/2, p-EGFR, p-AKT, p-STAT3, COX-2 and PGE2. A synergistic inhibitory effect was shown by combination treatment with Iressa (EGFR tyrosin kinase inhibitor) and SC-236 (COX-2inhibitor) in human breast and colon cancer lines and in mice bearing colon cancer xenograft [123]. Choe et al. showed the same results in HNSCC in vitro and in vivo by combination treatment with celecoxib and Iressa [124]. Preclinical combined treatment with erlotinib (EGFR tryrosin kinase inhibitor) and cetuximab showed significant inhibition in growth of head and neck cancer cell and animal xenograft model. Tissue biopsy in preclinical study among patients that received simultaneously erlotinib and cetuximab to showed a remarkable down regulate in pro-survival pathways such as ERK and AKT [125]. A clinical study among 107 patients with non-small cell lung cancer that were received erlotinib plus celecoxib or placebo demonstrated that progression-free survival was improved between patients receive both erlotinib and celecoxib as compared to placebo group [126]. A supra additive effect in inhibition of clonogenic survival, apoptosis enhancement and tumor growth suppression was observed by simultaneously combination treatment with celecoxib (COX-2I), erlotinib (EGFR inhibitor) and IR in the HNSCC and phase I clinical study [127].

## Conclusion and perspectives

**Fig. 5:**
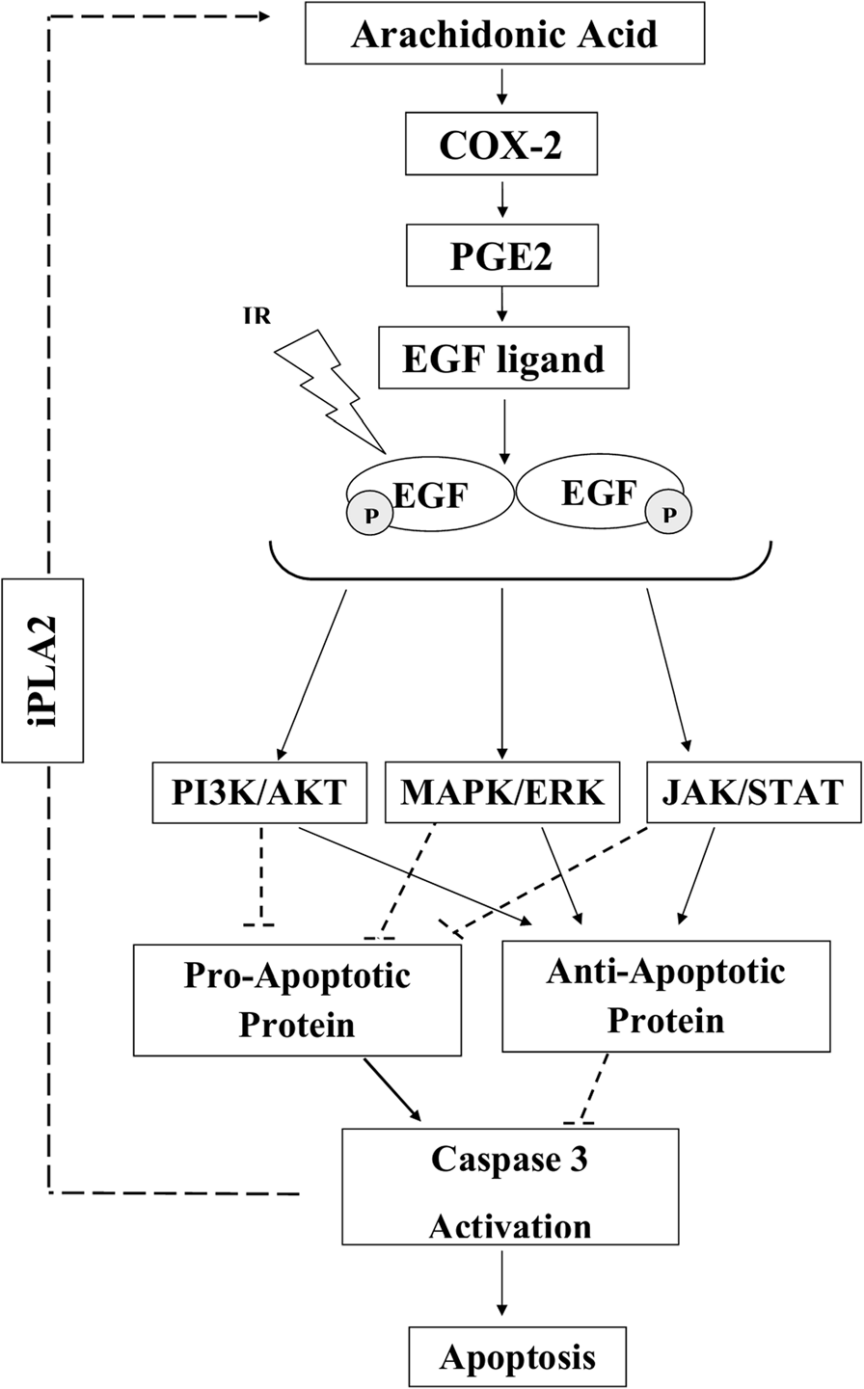
A schematic representation of the role of COX-2 (Cyclooxigenases-2) in proliferation of cancer cells. Radiation as well as PGE2 (Prostaglandin E2) are able to transactivate EGFR (Epidermal Growth Factor Receptor) that upregulates the level of pro- survival protein through PI3K/AKT, MAPK/ERK and JAK/STAT pathways. Also, caspase activation leads to release of arachidonic acid

Induction of apoptosis by IR and chemotherapeutic agents is a known strategy for killing human malignant cells. ROS that is produced during radiation therapy damage DNA and as a result trigger apoptotic pathway. Apoptosis induction through intrinsic and extrinsic pathways leads to caspase cascade activation. Also the mechanism of action of many radiosensitizers is based on caspase cascade activation. Caspase 3 act as an execuatior mediator in apoptosis, tumor repopulation through iPLA2 activation and PGE2 production. Repopulation of tumors results in failure of therapy. Release of PGE2 upregulates the level of expression of pro-survival proteins including: p-ERK1/2, p-EGFR, p-AKT, p-STAT3, COX-2 and PGE-2, which lead to apoptosis suppression and tumor proliferation. As a hypothesis, caspase 3 has a paradox role where its activation results in apoptosis and simultaneous acceleration of re-population of surviving tumor cells. Blockage of caspase 3 activity is a strategy to overcome radioresistance. On the other hand dependency between production of apoptosis mediator and increase in PGE2 and iPLA2 activity is a major reason for the treatment failure in radiotherapy. Results of investigations have shown that the use of COX-2 inhibitors may lead to more effective treatment. Furthermore the combination use of EGFR inhibitor and IR with COX-2 inhibitor is more impressive therapeutic regimen than the one without EGFR inhibitor. However, more clinical experiments are required to clarify the advantages of adjuvant use of cyclooxygenase, EGFR or caspase inhibitor in combination with radiotherapy (Fig. 5).

## Abbreviation

APAF-1: Adapter Protein Apoptotic protease-activating Factor-1
AVPI peptide: Ala-Val-Pro-Ile (AVPI) tetrapeptide
BIR3: Baculovirus IAP Repeat 3
c-IAP2: Baculoviral IAP repeat-containing protein3 (also known as cIAP2) is a protein that in humans is encoded by the BIRC3 gene
c-IAPl: Baculoviral IAP repeat-containing protein 2 (also known as cIAP1) is a protein that in humans is encoded by the BIRC2 gene
dATP: Deoxyadenosine triphosphate
DIABLO: Direct Inhibitor of Apoptosis protein-Binding protein with low pI. DIABLO is also referred to as second mitochondria-derived activator of caspases or SMAC.
DN_C3: dominant-negative mutants of caspase 3
DN_C7: dominant-negative mutants of caspase 7
DN_PKCδ: dominant-negative mutants of PKCd
ER: Endoplasmic reticulum
FADD: Fas-associating protein with a death domain
IAP: Inhibitors of apoptosis proteins
iPLA2: Ca(2+)-independent cytosolic phospholipase A2
lp-PLA2: lipoprotein-associated PLA2s
mTOR: MammalianTtarget of Rapamaycin
NAIP: Baculoviral IAP repeat-containing protein 1 is a protein that in humans is encoded by the NAIP gene
NF-kB: nuclear factor kappa-light-chain-enhancer of activated B cells
PGE2: Prostaglandin E2
PKCd: Protein kinase C delta type is an enzyme that in humans is encoded by the PRKCD gene
ROS: Reactive oxygen species
ULK complex: Serine/threonine-protein kinase ULK1 is an enzyme that in humans is encoded by the ULK1 gene
SMAC: Second mitochondria-derived activator of caspases
TNF: Tumor necrosis factor
TNFR1: Tumor necrosis factor receptor-1
TRAIL: TNF-relatedapoptosis-inducing ligand
XIAP: X-linked inhibitor of apoptosis protein (XIAP), also known as inhibitor of apoptosis protein 3 (IAP3) and baculoviral IAP repeat-containing protein 4 (BIRC), is a protein that stops apoptotic cell death. In humans, this protein (XIAP) is produced by a gene named XIAP gene located on the X chromosome
Z-DEVD: Caspase-3 inhibitor
